# Identification of cellular senescence-related genes and immune cell infiltration characteristics in intervertebral disc degeneration

**DOI:** 10.3389/fimmu.2024.1439976

**Published:** 2024-09-12

**Authors:** Muyi Wang, Hao Wang, Xin Wang, Yifei Shen, Dong Zhou, Yuqing Jiang

**Affiliations:** ^1^ Department of Orthopedics, Affiliated Changzhou Second People’s Hospital of Nanjing Medical University, Changzhou, Jiangsu, China; ^2^ Changzhou Medical Center, Nanjing Medical University, Changzhou, Jiangsu, China; ^3^ Department of Orthopedics, Affiliated Changzhou Children’s Hospital of Nantong University, Changzhou, Jiangsu, China

**Keywords:** intervertebral disc degeneration, cellular senescence, nucleus pulposus cells, consensus cluster analysis, immune infiltration

## Abstract

**Background:**

Intervertebral disc degeneration (IDD) progression involves multiple factors, including loss of nucleus pulposus cells and extracellular matrix as the basic pathological mechanism of degeneration, and is closely related to cellular senescence and immune cell infiltration. The aim of study was to identify critical cellular senescence-related genes and immune cell infiltration characteristics in IDD.

**Methods:**

Four datasets, including GSE70362, GSE112216, GSE114169, and GSE150408, were downloaded from the Gene Expression Omnibus database. The senescence-related genes were acquired from the CellAge Database and intersected with differentially expressed genes (DEGs) between IDD and control samples for senescence-related DEGs (SRDEGs). Protein-protein interaction (PPI) network analysis was performed to obtain ten hub SRDEGs. A consensus cluster analysis based on these hub genes was performed to divide the patients into clusters. The functional enrichment, and immune infiltration statuses of the clusters were compared. Weighted gene co-expression network analysis was used to identified key gene modules. The overlapping genes from key modules, DEGs of clusters and hub SRDEGs were intersected to obtain potential biomarkers. To verify the expression of potential biomarkers, quantitative polymerase chain reaction (qPCR) and immunohistochemistry were performed by using human intervertebral disc tissues.

**Results:**

In the GSE70362 dataset, a total of 364 DEGs were identified, of which 150 were upregulated and 214 were downregulated, and 35 genes were selected as SRDEGs. PPI analysis revealed ten hub SRDEGs and consensus cluster analysis divided the patients into two clusters. Compared to Cluster 2, Cluster 1 was highly enriched in extracellular matrix organization and various metabolic process. The level of Follicular T helper cells in the Cluster 1 was significantly higher than that in the Cluster 2. IGFBP3 and NQO1 were identified as potential biomarkers. The remaining 3 datasets, and the result of qPCR and immunohistochemistry showed that the expression levels of NQO1 and IGFBP3 in the degenerated group were higher than those in the control or treatment groups.

**Conclusion:**

Senescence-related genes play a key role in the development and occurrence of IDD. IGFBP3 and NQO1 are strongly correlated with immune infiltration in the IDD and could become novel therapeutic targets that prevent the progression of IDD.

## 1 Introduction

Low back pain (LBP), a prevalent public health issue impacting individuals globally, not only inflicts physical discomfort on patients but also imposes a significant economic burden on society (1). Intervertebral disc degeneration (IDD) is one of the most common lumbar degenerative diseases and recognized as the major cause of LBP. Although substantial progress has been achieved in the management of discogenic LBP, a great number of patients still respond badly to the long-lasting clinical therapy. With the goal of finding potential preventive methods, many research has been done to explore various pathological mechanism of IDD, such as DNA replication errors, metabolic disturbances, inflammation, and loss of functional cells and stem cells (2). The intervertebral disc is anatomically composed of the internal nucleus pulposus (NP), the peripheral annulus fibrosus (AF), and the cartilaginous endplates adjacent to vertebral bodies. The NP tissue of intervertebral disc plays a critical role in maintaining the physiological functions of the spine and its dysfunction is widely recognized as the pivotal element in the development of IDD (3).

Cellular senescence, which features the irreversible cessation of the cell cycle, is a fundamental mechanism that mediates age-related dysfunctions and chronic diseases such as IDD and osteoarthritis (4–6). It has been reported that enhanced apoptosis significantly contributes to reduce nucleus pulposus cells (NPCs) and extracellular matrix (ECM), but senescent cells also accumulate in the degenerative NP (7). Despite cell cycle transition arrest and proliferation cease, senescent cells are metabolically viable, and exhibit altered expression of various catabolic cytokines and degrading enzymes (8). By reducing cell viability and changing its microenvironment, excessive senescence is causally linked to the degradation of ECM and loss of hydrophilic matrix molecules in the degenerative intervertebral discs (9). In the advanced stage of degeneration, collapsed disc space and generalized structural deterioration could alter the disc biomechanics, leading to the continuously elevated inflammation levels and pain factors (10).

Additionally, in healthy intervertebral disc tissue, neither blood vessels nor nerve cells are typically detected within the NP. This anatomical structure makes the NP isolate from the development of immunological tolerance and become an immune‐privileged organ (11). However, the damage of physical barrier between the IVD and the immune system due to ruptured AF leads to exposure of NP to the immune cells in the bloodstream and triggers immune response. Various immune cells, including macrophages, T cells, B cells, and NK cells, are involved in IDD progression or disc herniation (12). These infiltrating immune cells secrete a large amount of proinflammatory cytokines (IL‐1β, TNF‐α, IL‐6, and IFN‐γ et al.), and aggravate immune activation and inflammatory reactions (12, 13). Currently, biological therapy targeting immune and inflammation modulation for IDD is still in its early stages. The role of molecular immunology and the relationship between the immune response and cellular senescence in the process of IDD are not fully understood. More in-depth exploration in immune-related inflammatory response should be conducted to improve IDD treatment.

In general, both immunological infiltration and cellular senescence are significant risk factors of IDD, and targeting these mechanisms may develop novel preventive and therapeutic approaches to improve the management and treatment of this disease. In this study, we employed an integrated bioinformatics and basic experiments strategy to uncover critical genes correlated with cellular senescence and alterations in the immunological infiltration in degenerative NP tissues. The findings of this study will provide fresh light on the molecular and cellular research of IDD. To the best of our knowledge, this is the first study to examine the impact of cellular senescence on the immunological infiltration landscape in NP tissues using consensus cluster analysis, providing insights into novel therapeutic strategies and a substantial theoretical foundation for future innovative studies.

## 2 Materials and methods

### 2.1 Data collection and preprocessing

The transcriptome profiling data for IDD were downloaded from four datasets in the Gene Expression Omnibus (GEO) database, including GSE70362, GSE112216, GSE114169, and GSE150408. Specifically, GSE70362 (based on the GPL17810 platform) contained 16 degenerated disc NP tissues and 8 control NP tissues. GSE112216 (GPL16686 platform) included 3 samples (degenerative NPCs co-cultured with adipose-derived mesenchymal stem cells (ASCs)) and 3 control (degenerative NPCs only). GSE114169 (GPL15314 platform) contained 4 samples (NPCs from lumbar disc herniation patients treated with neurotropin) and 4 control (NPCs from lumbar disc herniation patients). GSE150408 (GPL20301 platform) contained 17 peripheral blood samples from patients with lumbar disc herniation and 17 peripheral blood samples from healthy volunteers. The GSE70362 dataset was used to identify the differentially expressed genes (DEGs). The GSE112216, GSE114169, and GSE150408 datasets were used to validate the expression of senescence-related genes. The senescence-related genes were acquired from the CellAge online Database (https://www.genomics.senescence.info/cells).

The principal component analysis (PCA) of GSE70362 dataset was performed to check the sample separation between IDD and control groups by using the “FactoMineR” and “factoextra” packages in R. The “tinyarray” package was used to perform probe annotation for the microarray data. When a gene corresponded to multiple probe IDs, only the first ID will be preserved.

### 2.2 Determination of DEGs

The DEGs were acquired by utilizing the R package “limma”, and the criterions of identifying the DEGs were set as |log2FC| > 0.585 and P value < 0.05. The senescence-related DEGs (SRDEGs) were acquired by the intersection of DGEs based on GSE70362 and senescence-related genes based on CellAge database by using the Venn diagram. Volcano plot of the DEGs, and hierarchical cluster heatmap of the SRDEGs were obtained by the “ggplot2” and “pheatmap” R packages.

### 2.3 Functional enrichment analysis

The Gene Ontology (GO) and Kyoto Encyclopedia of Genes and Genomes (KEGG) pathway enrichment analyses were performed by using the clusterProfiler package in R. The GO classification system is composed of three categories: biological process, cell component, and molecular function. The analysis was restricted to the species “Homo sapiens”. The SRDEGs list was employed for GO and KEGG enrichment analysis. All gene symbols in the SRDEGs list were converted to ENTREZ IDs. Following this, the enrichGO and enrichKEGG functions were applied to acquire GO terms and KEGG maps of biological functions. A threshold P value of < 0.05 was considered statistically enriched. The R package “ggplot2” was used for the visualization of the enriched items.

### 2.4 Protein-protein interaction network analysis

The protein-protein interaction (PPI) network of the SRDEGs was established based on the STRING database v12.0 (https://cn.string-db.org/). The protein interaction pairs with a confidence score >0.40 were selected and further imported to the Cytoscape software v3.9.1. By using the MCC analysis method in the CytoHubba plug-in, and the top ten significantly connected nodes were selected as the hub SRDEGs for further analysis.

To evaluate the relationships among these hub SRDEGs, the correlations among the hub SRDEGs were conducted by using Pearson correlation analysis. P value < 0.05 imply that the genes have a strong correlation. The correlation matrix heatmap and thescatter plots were mapped by using the “corrplot”, “ggplot2”, and “ggstatsplot” R packages.

### 2.5 Consensus cluster analysis

The R package “ConsensusClusterPlus” was applied to classify NP samples of GSE70362 dataset into different clusters based on the hub SRDEGs. The maximum subtype k was 8 and the optimal clusters number was comprehensively evaluated based on the result of the cumulative distribution function (CDF) curve, consensus matrix and consistent cluster score (>0.9). The subtype assignments were checked using t-distributed stochastic neighbor embedding (tSNE) analysis. The R package “limma” was used again to analyze DEGs of clusters, with |log2FC| > 0.585 and P < 0.05 as the differential gene-screening criteria. The gene expression profiles of hub SRDEGs within different clusters also were compared and visualized using the R packages “ggpubr” and “ggplot2”. The GO enrichment analysis of clusters was performed by using the clusterProfiler package in R.

### 2.6 Weighted gene co-expression network analysis

The R package “WGCNA” was used to identify co-expression modules. The variability of gene expressions across the samples of GSE70362 dataset was measured by median absolute deviation (MAD) method, and the top 5000 MAD genes were identified for following processing. After removing outlier samples in the cluster tree by using the “flashclust” R package, residual samples were reserved for subsequent analysis. When a rational soft power threshold was determined through the “pickSoftThreshold” function, an adjacency matrix was constructed and converted into a topological overlap matrix (TOM). The TOM-based phase dissimilarity metric with a minimum gene volume of 30 for the gene dendrogram was utilized to categorize genes with similar expression patterns into gene modules using average linkage hierarchical clustering. The module with the strongest relevance to clusters form consensus cluster analysis were selected as key modules. The gene significance (GS) and module membership (MM) values of all genes within the module were also generated and the genes were selected when the GS > 0.7 and MM > 0.6. The overlapping genes from key module genes, DEGs of clusters and senescence-related genes were selected as cluster genes for further analysis.

### 2.7 Single-gene gene set enrichment analysis

The cluster genes and hub SRDEGs were intersected to obtain potential biomarkers for further analysis. To explore the significant pathways associated with potential biomarkers, the gseKEGG function [Gene Set Enrichment Analysis (GSEA) of KEGG] of the R “clusterProfiler” package was used. According to the expression value, the correlation coefficients of potential biomarkers with all genes in the gene sets were ranked. The threshold for enrichment significance was |NES| >1, and adjust P value was <0.05.

### 2.8 Construction of ceRNA and mRNA–transcription factor networks

A ceRNA network was constructed based on the identified potential biomarkers. To interact mRNAs and miRNAs in a reliable way, 3 validated databases (“mirecords”, “mirtarbase”, and “tarbase”) in the R package “multiMiR” was employed. To obtain the miRNA-targeted lncRNAs, the miRNA-Target data from ENCORI/starBase were downloaded (https://rnasysu.com/encori/index.php). To construct the ceRNA network, the mRNA-miRNA and miRNA-lncRNA pairs were visualized with Cytoscape software (version 3.9.1). To analyze the regulatory effect of transcription factors (TFs), the targeted relationships between TFs and potential biomarkers were retrieved from the Network Analyst database (https://www.networkanalyst.ca/), and an interaction network between potential biomarkers and TFs was constructed and visualized with Cytoscape software (version 3.9.1).

### 2.9 Immune infiltration analysis by CIBERSORT algorithm

Immune cell infiltrations of IDD and control samples were evaluated by using a bioinformatics algorithm called CIBERSORT. The R package “CIBERSORT” and the leukocyte gene signature matrix LM22 were used to simulate and calculate the transcription feature matrix of 22 immune cells, and the number of calculations was set to 1,000. The differences between the IDD and control samples in the percentage of immune cells were tested using the Wilcoxon test, with a P value < 0.05 considered to be statistically significant. The “tinyarray” R package was used to visualize the differences between the immune cells of IDD and control groups. The correlations between immune cells and potential biomarkers, as well as the correlations between different immune cell, were calculated using the “corrplot” R package and Pearson correlation analysis.

### 2.10 External validation of potential biomarkers expression

The mRNA expression of identified potential biomarkers was verified in GSE56081, GSE112216, and GSE176205. The comparison between the two sets of data was performed with the Wilcoxon test. P < 0.05 was considered significant. Quantitative Polymerase Chain Reaction (qPCR) was performed to analyze the mRNA expression of potential biomarkers in IDD and control samples. This study was performed in line with the principles of the Declaration of Helsinki and approved by the ethics committee of our hospital. Informed consent was obtained from all individual participants included in the study. The degree of NP was determined by magnetic resonance imaging (MRI) following Pfirrmann classification (14). Tissues of Pfirrmann I-II were assigned to the control/non-degenerative group, whereas NP samples with grade III–V were assigned to the degenerative group. Human NP tissues were obtained from patients who underwent posterior spinal fusion surgery. Total RNA extraction and RNA reverse transcription were done as described in our previously report (15). A 7500 Real-time PCR system (Applied biosystems) was used to performed qPCR analyses. GAPDH was set as an internal control. All primers were listed in **Supplementary Table S1**.

NP tissues were fixed in 4% paraformaldehyde, then dehydrated, and embedded in paraffin. About 5 μM-thick sections were made for immunohistochemistry. After deparaffinization, tissue sections were incubated overnight at 4°C with NQO1 (11451-1-AP, proteintech, China) and IGFBP3 (10189-2-AP, proteintech, China) antibodies after epitope retrieval, H_2_O_2_ treatment, and non-specific antigens blocking. Then the sections were treated for two hours at room temperature with secondary antibodies, and an enhanced DAB staining kit was used to detect the signal. The ImageJ software was used for histomorphometric evaluation.

### 2.11 Statistical analyses

In the current study, R 4.3.1, SPSS version 25.0, GraphPad Prism 8 and Cytoscape software version 3.9.1 were used for data processing, statistical analysis, and plotting of graphs. To compare two normally distributed continuous variables, an independent samples t-test was employed. The Wilcoxon test was utilized to evaluate differences among variables that did not follow a normal distribution. A P value < 0.05 was considered statistically significant.

## 3 Results

### 3.1 PCA analysis and SRDEGs

**Figure 1:**
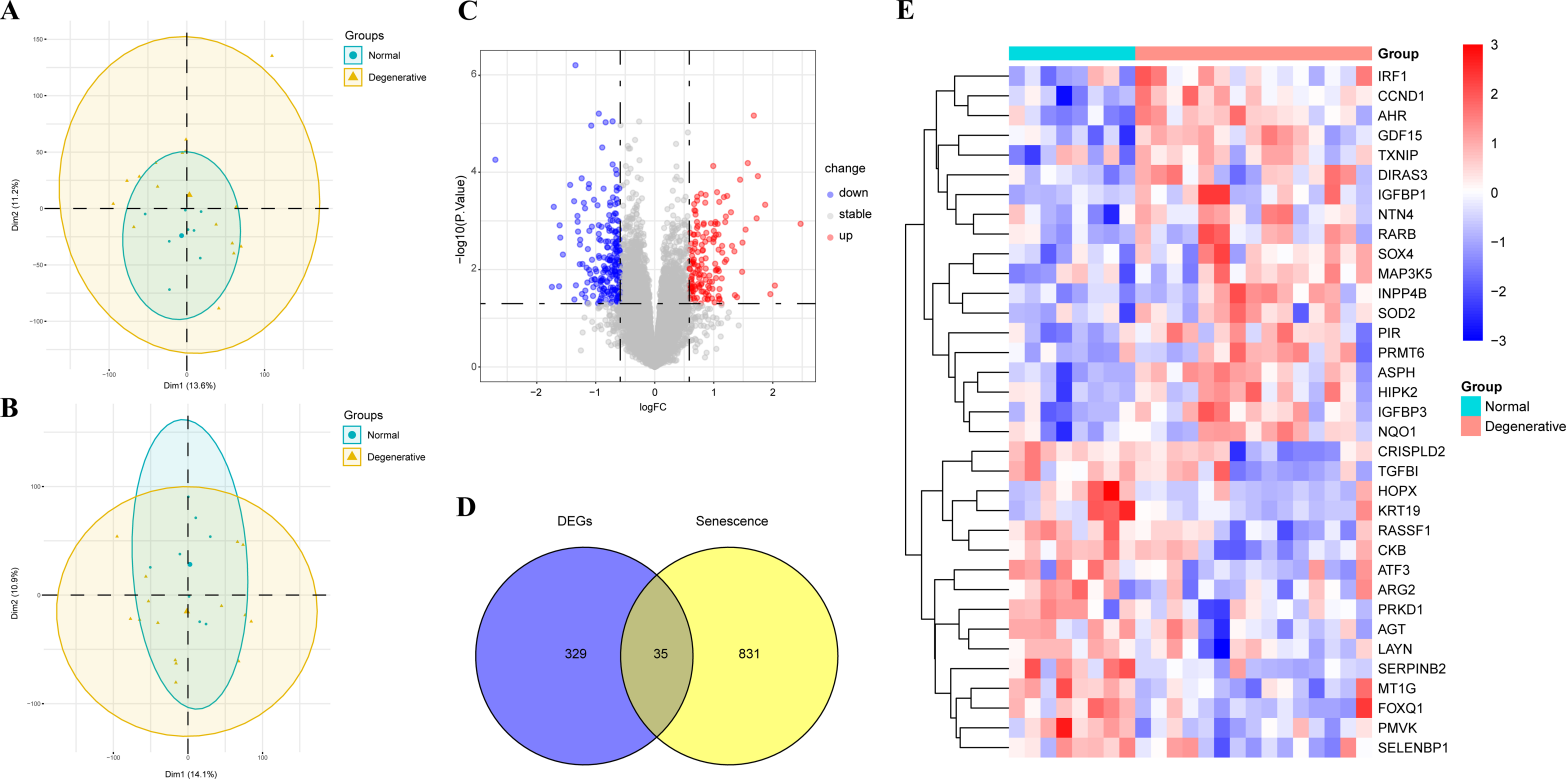
PCA analysis and identification of SRDEGs in IDD and controls. **(A)** PCA analysis before removing the outlier; **(B)** PCA analysis after removing the outlier; **(C)** Volcano map of DEGs between IDD and control samples; **(D)** Venn diagram of SRDEGs between DEGs and senescence-related genes; **(E)** Heat map of 35 SRDEGs in the IDD and control samples. PCA, Principal component analysis; IDD, intervertebral disc degeneration; DEGs, differentially expressed genes; SRDEGs, senescence-related differentially expressed genes.

After conducting PCA analysis on the GSE70362 dataset, it was observed that one sample deviated significantly from the overall clustering pattern, indicating its potential as an outlier (**Figure 1A**). To minimize the impact of this outlier on the subsequent analysis, it was decided to remove this specific sample from the dataset. By removing the outlier, the subsequent PCA analysis was performed on a refined dataset that better reflected the true clustering patterns of the remaining samples (**Figure 1B**).

A total of 364 DEGs were identified using the “limma” package with P < 0.05, of which 210 were downregulated and 154 were upregulated. The volcano plots of GSE70362 were shown in **Figure 1C**. By overlapping the 364 DEGs with 866 SRDEGs, 35 SRDEGs that differed significantly between the IDD and control groups were identified (**Figure 1D**). The gene expression patterns of 35 SRDEGs were presented in the heatmap (**Figure 1E**).

### 3.2 Functional enrichment analysis

**Figure 2:**
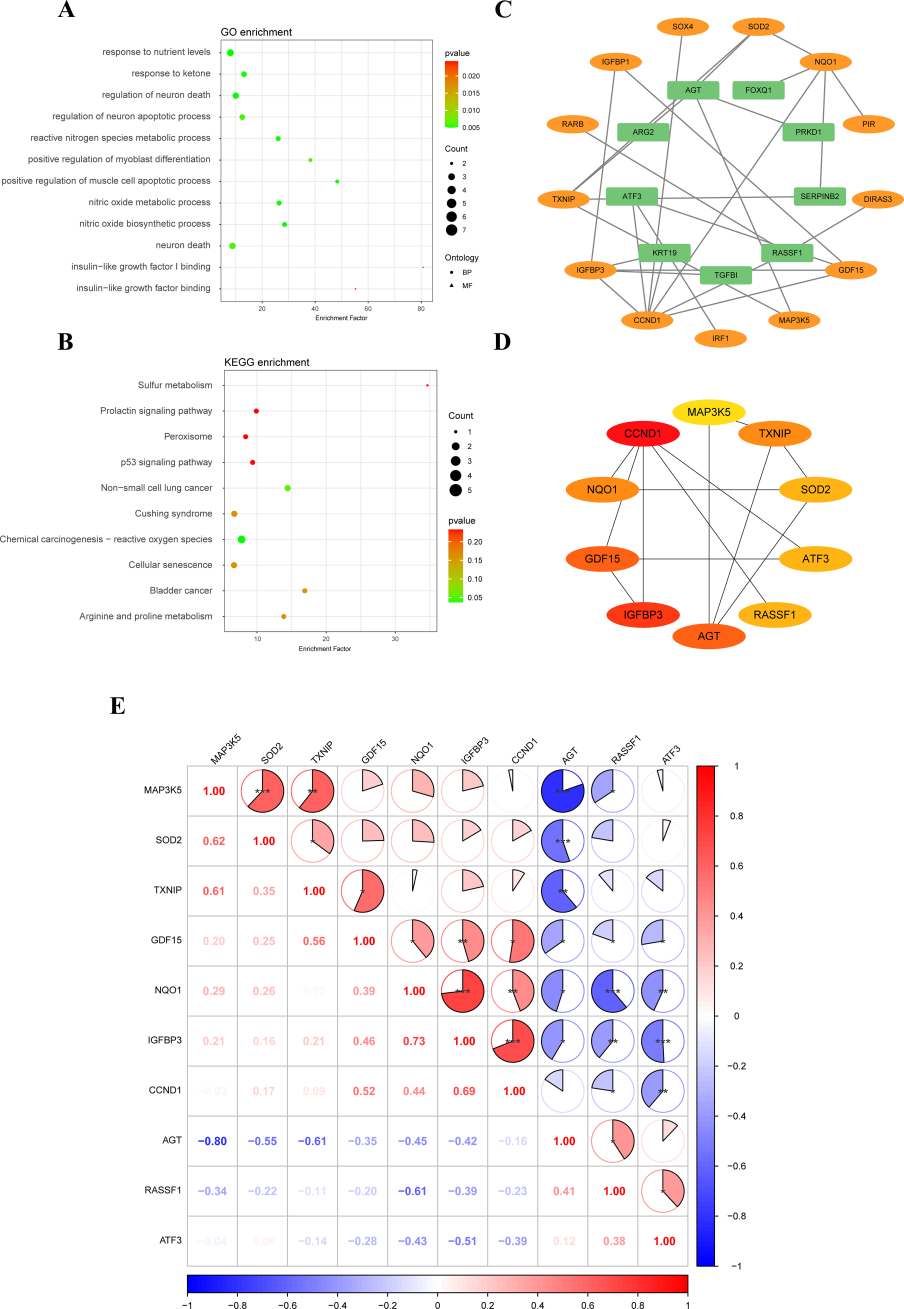
Functional enrichment analysis and construction of PPI network. **(A)** GO enrichment analysis of 35 SRDEGs; **(B)** KEGG pathway enrichment analyses of SRDEGs; **(C)** PPI network construction of 35 SRDEGs; **(D)** The top 10 SRDEGs using MCC analysis; **(E)** Correlation of SRDEGs. PPI, protein-protein interaction; SRDEGs, senescence-related differentially expressed genes; GO, Gene Ontology; KEGG, Kyoto Encyclopedia of Genes and Genomes. *represents P < 0.05, **represents P < 0.01, ***represents P < 0.001.

GO enrichment analysis showed that 35 SRDEGs were primarily enriched in biological process, including nitric oxide biosynthetic process, nitric oxide metabolic process, reactive nitrogen species metabolic process, response to nutrient levels, regulation of neuron death, positive regulation of muscle cell apoptotic process, response to ketone, regulation of neuron apoptotic process, neuron death, and positive regulation of myoblast differentiation. In molecular function, these genes were mainly enriched in insulin-like growth factor binding, and insulin-like growth factor I binding (**Figure 2A**; **Supplementary Table S2**). KEGG enrichment analysis showed that 35 SRDEGs were significantly activated in the cellular senescence, chemical carcinogenesis - reactive oxygen species, and p53 signaling pathway (**Figure 2B**; **Supplementary Table S3**).

### 3.3 PPI network construction

The PPI network analysis was performed based on the STRING database and visualized by Cytoscape software (**Figure 2C**). Then the top ten hub SRDEGs were determined based on the PPI score, including CCND1, ATF3, IGFBP3, GDF15, MAP3K5, TXNIP, RASSF1, AGT, SOD2, and NQO1, as shown in **Figure 2D**. Among these hub SRDEGs, ATF3, AGT, and RASSF1 were downregulated in IDD, and the rest seven genes were obviously upregulated. Then, Pearson correlation analysis was performed to evaluate the relationships among these hub SRDEGs (**Figure 2E**). The most negatively related pair was MAP3K5-AGT (r= -0.80 and P < 0.01), and the most positively related pairs were NQO1-IGFBP3 (r= 0.73 and P < 0.001) and IGFBP3-CCND1 (r= 0.69 and P < 0.001).

### 3.4 Consensus cluster analysis

**Figure 3:**
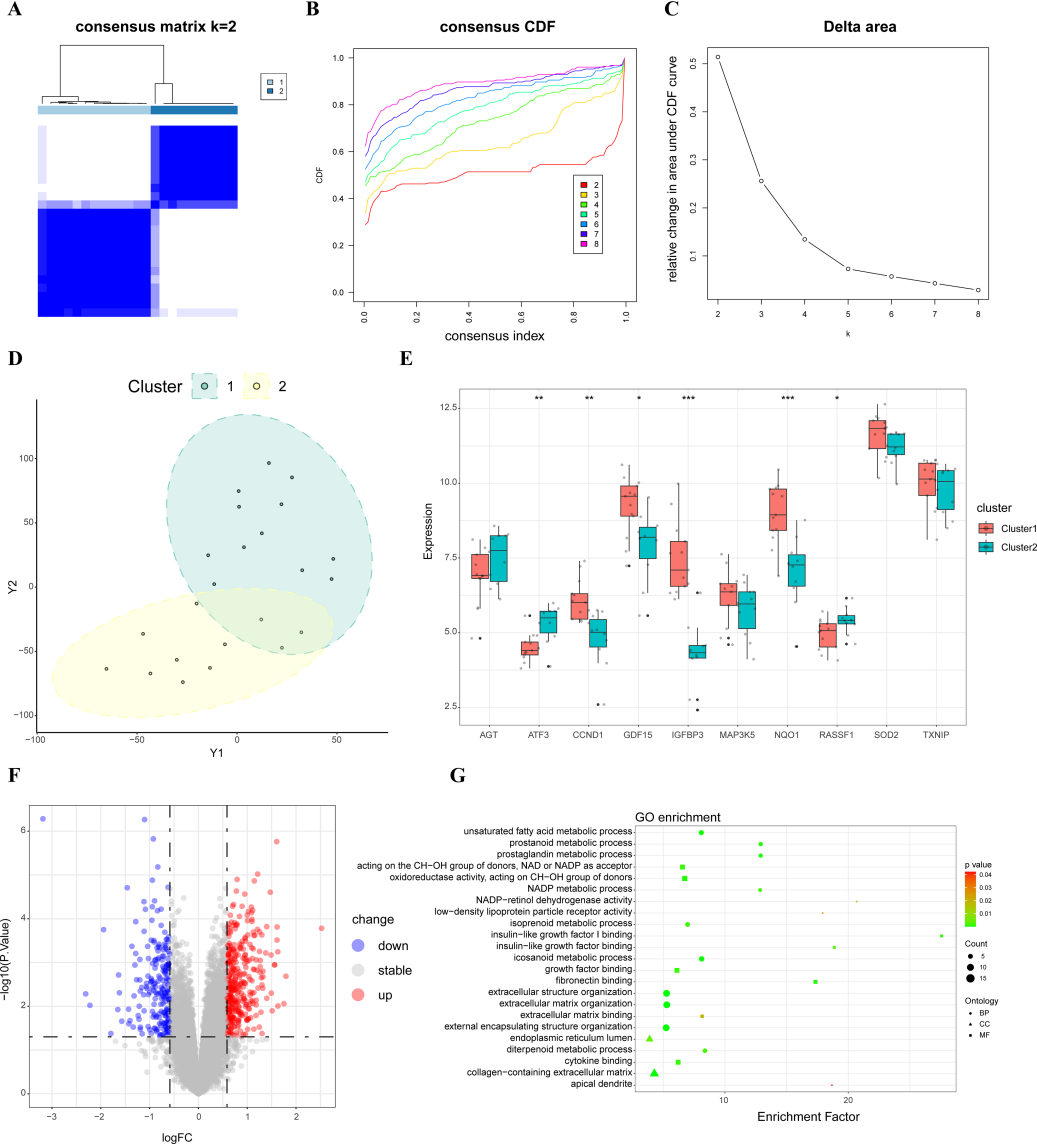
Consensus cluster analysis. **(A)** Consensus matrix when k=2; **(B)** Representative CDF curves; **(C)** CDF delta area curves when k was ranged 2 to 8; **(D)** tSNE analysis of two clusters; **(E)** Hub SRDEGs expression between two clusters; **(F)** Volcano map of DEGs between two clusters; **(G)** GO enrichment analysis between two clusters. CDF, cumulative distribution function; tSNE, t-distributed stochastic neighbor embedding; DEGs, differentially expressed genes; SRDEGs, senescence-related differentially expressed genes; GO, Gene Ontology. *represents P < 0.05, **represents P < 0.01, ***represents P < 0.001.

Using an unsupervised consensus clustering technique, 23 samples were classified based on the expression of top ten hub SRDEGs. The consensus matrix analysis revealed that k=2 was the ideal choice (**Figures 3A–C**). Cluster 1 contained 13 samples and Cluster 2 contained 10 samples. The tSNE analysis revealed a distinction between the two clusters (**Figure 3D**). The expression level of CCND1, GDF15, IGFBP3, and NQO1 was significantly higher in Cluster 1 than those in Cluster 2, whereas the expression level of ATF3 and RASSF1 was higher in Cluster 2 than that in Cluster 1 (**Figure 3E**). A total of 573 DEGs were identified between Cluster 1 and Cluster 2. The volcano plots were shown in **Figure 3F**. The result of GO enrichment analysis showed that Cluster 1 mainly enriched in biological process, such as extracellular matrix organization, extracellular structure organization, and cell component, such as collagen-containing extracellular matrix, endoplasmic reticulum lumen, as well as molecular function such as insulin-like growth factor binding, fibronectin binding. (**Figure 3G**; **Supplementary Table S4**).

### 3.5 WGCNA analysis

**Figure 4:**
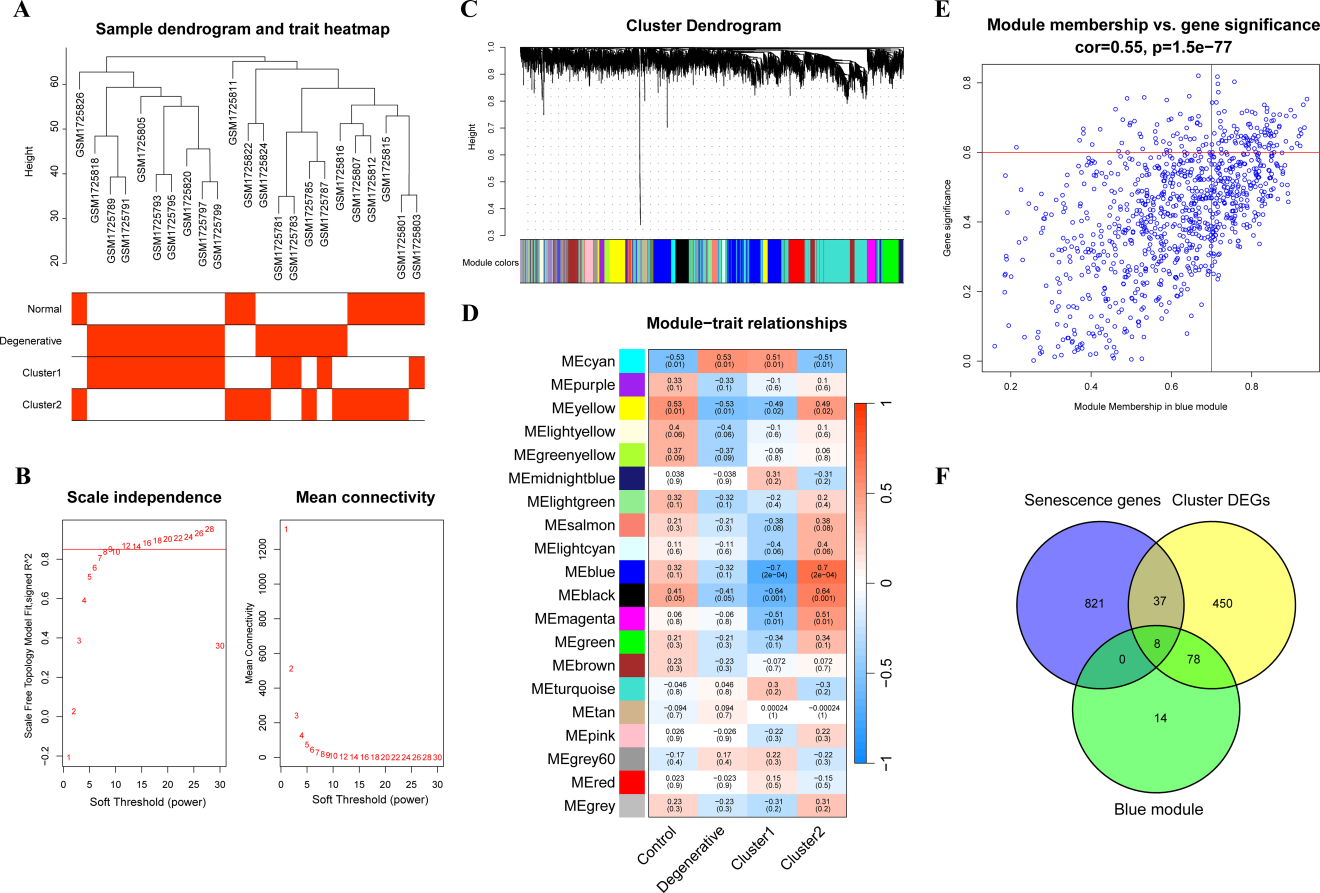
WGCNA analysis. **(A)** The sample dendrogram and feature heat map; **(B)** Graphs of scale independence, mean connectivity, and scale-free topology; **(C)** Gene clustering dendrogram with dynamic identification of modules; **(D)** Module-trait relationships; **(E)** Scatter plot analysis of blue module, key module genes were screened out in the upper-right area where GS > 0.7 and MM > 0.6; **(F)** Venn diagram of cluster genes among key module genes, DEGs of clusters and senescence-related genes. WGCNA, weighted gene co-expression network analysis; GS, gene significance; MM, module membership; DEGs, differentially expressed genes.

To further precisely excavate key genes associated with senescent clusters, a gene co-expression network using the WGCNA algorithm was constructed. The sample hierarchical cluster analysis results showed good clustering among the samples, with no significant outliers (**Figure 4A**). The soft threshold was set to 9 to satisfy the scale-free topology of the network, where the corresponding R^2^ was 0.85 and the average connectivity was high (**Figure 4B**). A gene hierarchy clustering dendrogram was constructed by gene correlation, and a total of 20 gene modules were identified (**Figure 4C**). The blue module, containing 967 genes, exhibited the strongest correlation with the senescent clusters (**Figure 4D**). The scatter plot (**Figure 4E**) showed a strong correlation between GS and MM in the blue module. Finally, 100 module genes were screened out for the subsequent analysis by setting the thresholds of GS > 0.6 and MM > 0.7. Then, eight genes, AKR1B1, ALKBH3, HIPK2, IGFBP3, NQO1, PRKCH, RARB and SAT2, were selected as the cluster genes (**Figure 4F**).

### 3.6 Single-gene GSEA and construction of networks

**Figure 5:**
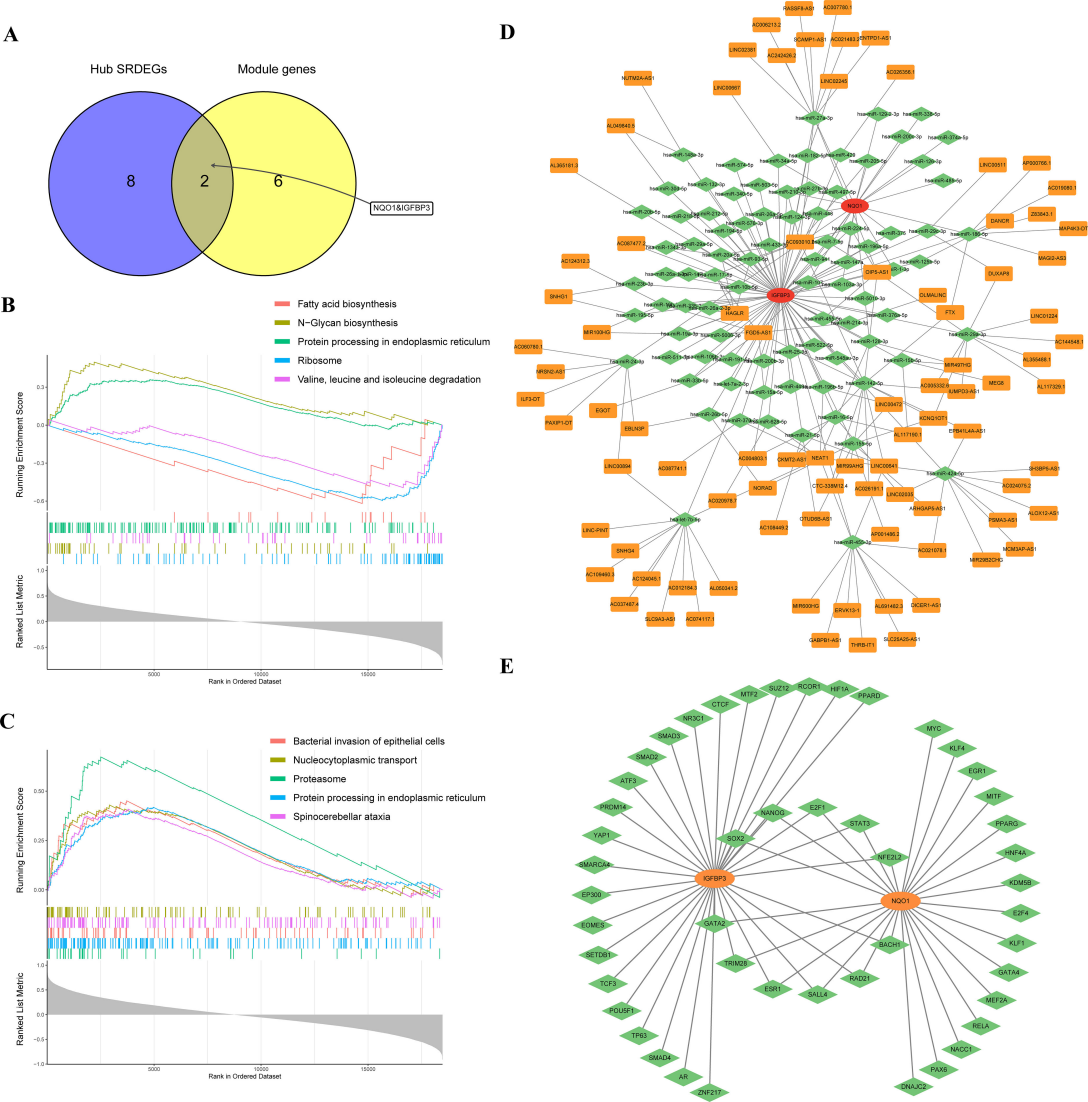
Single-gene GSEA and construction of ceRNA and mRNA–TF networks. **(A)** Venn diagram of overlapping genes between hub SRDEGs and module genes; **(B)** single-gene GSEA of IGFBP3; **(C)** single-gene GSEA of NQO1; **(D)** ceRNA network; **(E)** mRNA–TF network. GSEA, gene set enrichment analysis; TF, transcription factor; SRDEGs, senescence-related differentially expressed genes.

The cluster genes and hub SRDEGs were further intersected to obtain 2 potential biomarkers, IGFBP3 and NQO1 (**Figure 5A**). Subsequently, single-gene GSEA were performed to explore the biological functions and potential pathways associated with two biomarkers. The top five KEGG pathways, “Ribosome”, “Valine, leucine and isoleucine degradation”, and “Fatty acid biosynthesis” were negatively correlated with IGFBP3. “N-Glycan biosynthesis” and “Protein processing in endoplasmic reticulum” were positively correlated with IGFBP3 (**Figure 5B**; **Supplementary Table S5**). For NQO1, “Proteasome”, “Protein processing in endoplasmic reticulum”, “Spinocerebellar ataxia”, “Bacterial invasion of epithelial cells”, and “Nucleocytoplasmic transport” were activated most significantly (**Figure 5C**; **Supplementary Table S6**). We also noted that twenty-five pathways were correlated with both potential biomarkers, such as “Necroptosis”, “Alzheimer disease”, “Regulation of actin cytoskeleton”, “TGF-beta signaling pathway” and “Cellular senescence”. Based on the two potential biomarkers, ceRNA and mRNA–TF networks were developed. The ceRNA network included 239 interactions, 86 miRNAs, and 89 lncRNAs (**Figure 5D**; **Supplementary Table S7**). The mRNA-TF network included 59 interactions and 48 TFs. NQO1 interacted with 26 TFs, while IGFBP3 interacted with 33 TFs (**Figure 5E**; **Supplementary Table S8**).

### 3.7 Immune infiltration analysis

**Figure 6:**
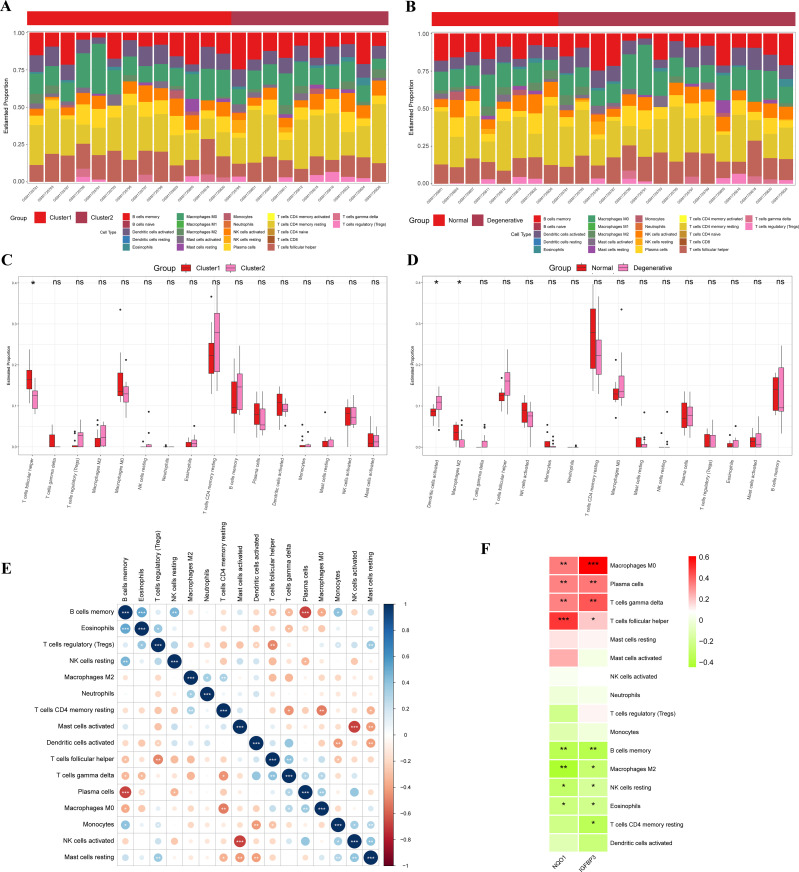
Immune infiltration analysis. **(A)** Accumulation diagram of immune cell content between Cluster 1 and Cluster 2; **(B)** Accumulation diagram of immune cell content between IDD and control samples; **(C)** Histogram of the content of immune cells in Cluster 1 and Cluster 2; **(D)** Histogram of the content of immune cells in IDD and control samples; **(E)** Correlation of immune cells; **(F)** Correlation between two potential biomarkers and immune cells. IDD, intervertebral disc degeneration. *represents P < 0.05, **represents P < 0.01, ***represents P < 0.001.

The results of the CIBERSORT analysis showed that the level of Follicular T helper (Tfh) cells in the Cluster 1 was significantly higher than that in the Cluster 2 (**Figures 6A, C**). Also, IDD group had more activated dendritic cells and less M2 macrophages than the control group (**Figures 6B, D**). The interaction between immune cells was visualized in **Figure 6E**. The results demonstrated that memory B cells had a significant negative correlation with plasma cells, while they had a significant positive correlation with Eosinophils. Regulatory T cells (Tregs) had a significant negative correlation with TFH cells but a significant positive correlation with resting mast cells. Activated NK cells had a significant negative correlation with activated mast cells but a significant positive correlation with monocytes.

Pearson correlation analysis was applied to examine the relationship between the two potential biomarkers and infiltrating immune cells. The results indicated that both NQO1 and IGFBP3 had a significant negative correlation with memory B cells, M2 macrophages, resting NK cells, and Eosinophils, while they had a significant positive correlation with M0 Macrophages, plasma cells, Gammadelta T cells (γδ T cells), and Tfh cells (**Figure 6F**). Additionally, IGFBP3 had a significant negative correlation with resting memory CD4^+^ T cells.

### 3.8 External validation

**Figure 7:**
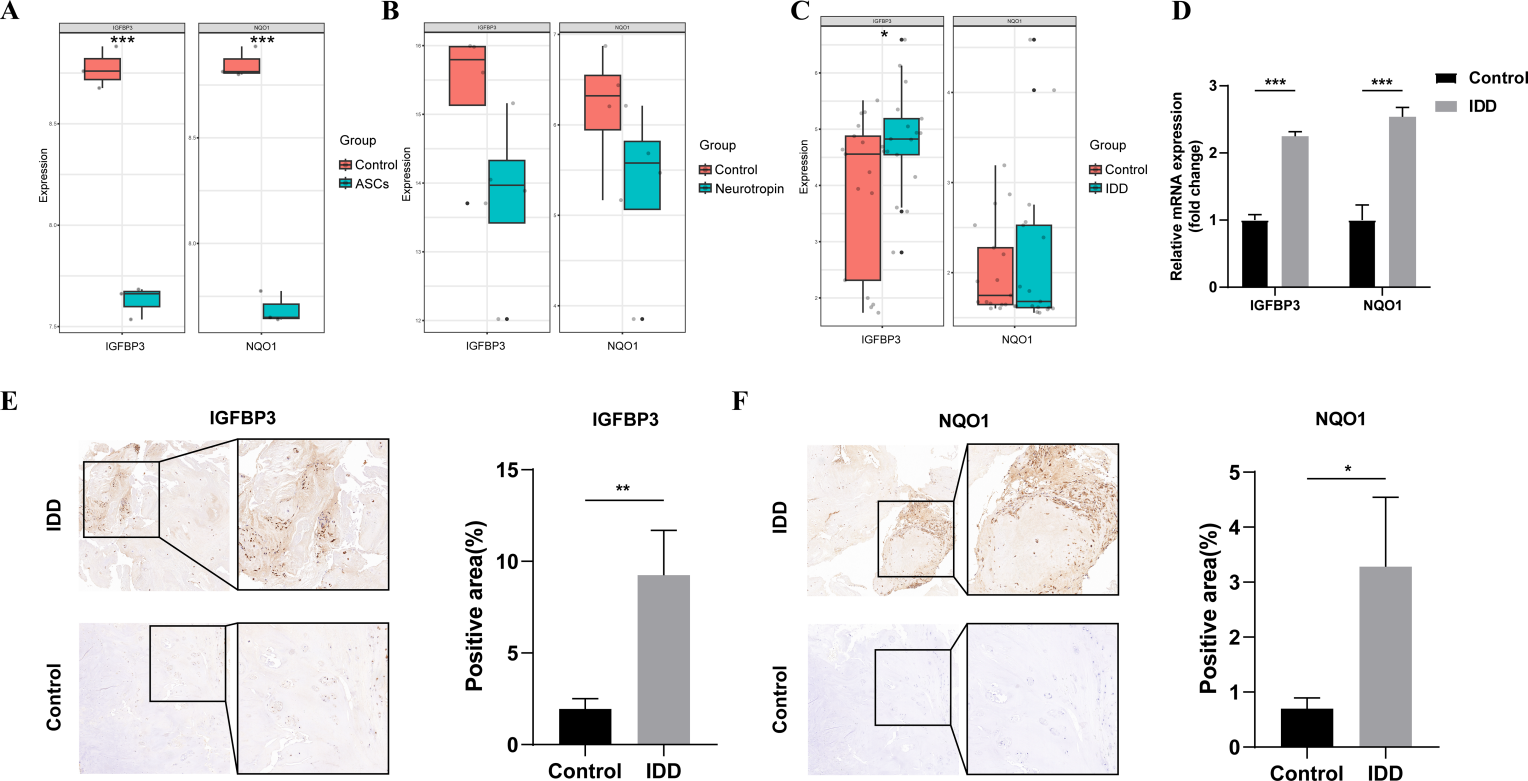
External validation. **(A)** mRNA expression levels of NQO1 and IGFBP3 between ASCs and control groups in the GSE112216 dataset; **(B)** mRNA expression levels of NQO1 and IGFBP3 between neurotropin and control groups in the GSE114169 dataset; **(C)** mRNA expression levels of NQO1 and IGFBP3 between IDD and control groups in the GSE150408 dataset; **(D)** mRNA expression levels of NQO1 and IGFBP3 between IDD and control samples; **(E)** Immunohistochemical staining of IGFBP3 and histograms of quantitative immunohistochemical staining results; **(F)** Immunohistochemical staining of NQO1 and histograms of quantitative immunohistochemical staining results. ASCs, adipose-derived mesenchymal stem cells; IDD, intervertebral disc degeneration. *represents P < 0.05, **represents P < 0.01, ***represents P < 0.001.

To verify the expression of potential biomarkers, the differential expression analyses were further performed in the GSE112216, GSE114169, and GSE150408 datasets. In the GSE112216 dataset, the expression level of IGFBP3 and NQO1 in the degenerative NPCs was decreased significantly after cocultured with ASCs (**Figure 7A**). Similarly, in the GSE114169 dataset, neurotropin treatment inhibited the expression of IGFBP3 and NQO1, but the differences did not reach a significant level (**Figure 7B**). We also found that IGFBP3 was differentially expressed in the peripheral blood of patients with lumbar disc herniation compared with that of healthy controls (**Figure 7C**). Then, 6 human NP tissues including 3 from patients with discs of Pfirrmann level II and 3 from patients with degenerated discs of level IV were collected (**Supplementary Table S9**). The result of qPCR showed that expression level of NQO1 and IGFBP3 in the degenerated group was significantly higher than those in the control group (**Figure 7D**). The immunohistochemistry also indicated a significant increase in the expression level of NQO1 and IGFBP3 in the NP tissues of IDD (**Figures 7E, F**).

## 4 Discussion

IDD is the most common chronic degenerative disease encountered in orthopedic clinics, among which the most common affected site is the lumbar spine. Despite years of efforts and attempts, the mechanisms of IDD remain controversial and there are no reports confirming that existing treatment measures can prevent or reverse progression of IDD. Clinical evidences suggest a close relationship between cell aging and worse outcomes in IDD patients (16, 17). There are also evidences that IDD is closely related to immune inflammation (2, 12). This provides new ideas and entry points for the treatment of IDD. However, although some biomarkers have been identified in previous studies, there is no focus on the comprehensive investigation of cellular senescence and immune infiltration related to the occurrence and development of IDD. Thus, this study was performed to explore potential SRDEGs of IDD and further investigate their relationship with immune infiltration based on comprehensive analysis.

We screened out the DEGs in IDD according to the public dataset, including 210 downregulated genes and 154 upregulated genes. Then we found that the expression of senescence-related DEGs in IDD group was different from that in control group. We identified 35 SRDEGs, including 16 downregulated SRDEGs and 19 upregulated SRDEGs. These genes were primarily associated with regulation of apoptotic process, metabolic process, and insulin-like growth factor binding. Consistent with this finding, features of cell senescence are frequently described by irreversible proliferation arrest, resistance to apoptosis, and enhanced catabolic metabolism (7, 18). Moreover, these genes were also involved in pathways, such as cellular senescence, p53 signaling pathway, and reactive oxygen species (ROS). Both p53 pathway and ROS play a critical role in aging, and their dysregulation is closely implicated in degenerative NP and could be a leading driving factor for the progress of IDD (19).

Immune cell infiltration analysis showed that the expression level of M2 macrophages was higher in normal group, whereas the expression level of activated dendritic cells was significantly higher in degenerative group. We speculated that this difference in immune cell infiltration may be related to the different IDD progression in the two groups. In the degenerative disc, disruption of ECM barrier and release of specific surface antigens from NPCs stimulate the corresponding dendritic cells of the immune system, trigger the process of cell-mediated immunity, and expand tissue damage and systemic inflammation (12). Also, low level of M2 macrophages in the degenerative group suggested that the patients had progressed to the stage of inflammatory circulation. Increasing evidences support that macrophages M1 polarization shows a proinflammatory effect, while M2 state plays an anti-inflammation and remodeling effect in response to injury (20).

Then, we conducted the PPI network analysis to further identify hub genes among these SRDEGs. We determined ten most important hub SRDEGs, as follows: CCND1, ATF3, IGFBP3, GDF15, MAP3K5, TXNIP, RASSF1, AGT, SOD2, and NQO1. To subdivide different characteristics of cell senescence, we attempted to use ten hub genes to perform a consensus cluster analysis on 23 NP samples and finally obtained two clusters, including 13 samples for Cluster 1 and 10 samples for Cluster 2. The expression of CCND1, ATF3, IGFBP3, GDF15, RASSF1, and NQO1 in both clusters was significantly increased or decreased. We investigated biologically related functions of two clusters and found that compared to Cluster 2, Cluster 1 was highly enriched in ECM organization and various metabolic process. These results suggested that Cluster 1 was significantly correlated with enhanced catabolic metabolism of cellular senescence, and Cluster 2 could be thought as a normal cluster.

Similarly, immune cell infiltration analysis also showed that the expression level of Tfh cells was higher in Cluster 1 than Cluster 2. This reflected different immune microenvironments of two clusters. NPCs aging plays a promoting role during IDD occurrence and development. At this stage, senescent NPCs could produce a large amount of pro-inflammatory factors, including IL-6, IL-1β, and TNF-α. These inflammatory chemokines will further recruit immune cells to infiltrate into the intervertebral disc (6, 19). Tfh cells are specialized providers of T cell help to B cells, and are essential for germinal center formation, affinity maturation, and the development of most high-affinity antibodies and memory B cells (21). The production of Tfh cells requires Bcl-6. IL-6, IL-12, and IL-27 activate transcription factors STAT3 or STAT4 to induce Bcl-6 expression and promote Tfh lineage differentiation (21, 22). There is currently no research reporting the role of Tfh cells in IDD. Previous studies have demonstrated that robust regulation of Tfh cell response and subsequent antibody maturation are critical for infection clearance, whereas aberrancy in controlling Tfh immune response is implicated in progression of autoimmune diseases such as systemic lupus erythematosus, arthritis, and type I/II diabetes (22). Therefore, the infiltration of Tfh cells within NP tissues may exacerbate the inflammatory response, and accelerate the aging of NPCs and disc degeneration. Further researches are needed to elucidate the regulatory role of Tfh cells in the process of cellular senescence.

Then, a combination of DEGs and WGCNA method was used to identify 2 potential biomarkers, IGFBP3 and NQO1. Subsequent single-gene GSEA analysis confirmed that these two genes significantly correlated with cell senescence, apoptosis, and some neurodegenerative diseases. The external validation also confirmed that the expression level of two genes significantly decreased in the NPCs of degenerative intervertebral disc after cellular or drug treatment. Meanwhile, we also found a significant increase in the expression of IGFBP3 in the peripheral blood of lumbar disc herniation patients compared to normal individuals. Also, immune infiltration analysis also found that these two genes were significantly correlated with various immune infiltration cells such as T cells, macrophages, and B cells. The above results indicated that cellular senescence and immune infiltration were closely related and had a synergistic effect on the progression of IDD, and these two genes may play a key role in the regulatory process.

IGFBP3 (Insulin-like growth factor binding protein 3), one of the six members of the insulin-like growth factor binding protein family, is a key protein in the insulin-like growth factor (IGF) pathway. IGFBP3 can function in an IGF-dependent as well as in an IGF-independent manner. The IGF-dependent roles of IGFBP3 include its endocrine role in the delivery of IGFs from the site of synthesis to the target cells that possess IGF receptors and the activation of associated downstream signaling (23). IGF-independent roles of IGFBP3 include its interactions with proteins of ECM and plasma membrane, and its translocation through plasma membrane into cytoplasm and nucleus (24). IGFBP3 is a well-documented inhibitor of cell growth and/or promoter of apoptosis in several cell types, primarily through the attenuation of IGF pathway (25). Also, these bioactivities can occur in the IGF-independent pathways. Evidences suggest an interaction between IGFBP3 and TGF-β signaling pathways. It has been demonstrated that growth inhibitory signal of IGFBP3 may require an active TGF-β signaling pathway and implicate Smad 2/3 in its signal transduction (24). Furthermore, IGFBP3 has been proposed as a functional ligand for TGF-β receptor V, which is characterized to inhibit cell growth (23).

Kim et al (26). suggested a potential role of IGFBP3 in the senescence of human umbilical vein endothelial cells, as downregulation of IGFBP3 by siRNA rescued the growth arrested induced by p53 overexpression. Moreover, an inverse correlation with Foxo3a activity was identified when overexpression of IGFBP3 accelerated senescence. Foxo3a belongs to the family of Forkhead transcription factors that appear to transcriptionally up-regulate antioxidant defenses, including superoxide dismutase and catalase. Foxo3a protein levels were increased in aged cells following IGFBP3 knockdown. Downregulation of Foxo3a is known to accelerate senescence in human dermal fibroblasts, suggesting a possible relationship between IGFBP3 and Foxo3a. However, the role of IGFBP3 in regulating IDD and NPCs aging is still unclear. Considering that IGFBP3 is one of the most highly expressed IGF binding proteins, and its role in regulating cell senescence has been demonstrated in certain cell types, we speculate that IGFBP3 may play a crucial role in regulating inflammation, immune response, oxidative stress, and cell senescence within the intervertebral disc. Therefore, in the upcoming research, we aim to delve deeper into the role and regulatory mechanisms of IGFBP3.

NQO1 (NAD(P)H:quinone oxidoreductase 1) is a cytosolic reductase that plays an important role in cellular responses to oxidative stress. NQO1 protects cells against various cytotoxic quinones and oxidative stress, and catalyzes reduction and detoxification of quinone substrates, thereby preventing cytotoxic effects of carcinogens (27). As a crucial anti-oxidative enzyme, NQO1 is likely to play a significant role in the intervertebral disc because oxidative stress contributes to the progress of IDD. Previous studies have reported that NQO1 declines with aging, providing less efficient protection against oxidative stress at many levels (28). Overexpression of a quinone reductase homolog in yeast extends both chronological and replicative lifespan (29). A recent high throughput anti-aging drug screen identified 2 compounds from more than 2600 screened, both of which affected pyridine nucleotide redox ratios and one of these compounds functioned via NQO1 catalysis (30, 31).

NQO1 also plays an important role in cancer. Numerous human cancers express 5- to 200-fold higher levels of NQO1 than their healthy tissue counterparts (32). Liu et al (33). reported that expression of NQO1was induced during oncogene-induced senescence (OIS). Depletion of NQO1 resulted in the delayed onset of senescence, while ectopic expression of NQO1 enhanced the senescence phenotype. Analysis of the mechanism underlying the up-regulation of NQO1 expression during senescence identified that NQO1 promoted p53 accumulation in an MDM2 and ubiquitin independent manner, which reinforced the cellular senescence phenotype. They also demonstrated that NRF2/KEAP1 signaling regulated NQO1 expression during OIS. Depletion of NQO1 facilitated cell transformation and tumorigenesis, which indicated that NQO1 took part in the senescence barrier and had anti-oncogenic properties in cell transformation (33). However, involvement and function of NQO1in IDD are less well understood than its role in cancer. From the above studies, we can see that NQO1 may have different roles in different cells and microenvironments. We believe that NQO1 may protect NPCs from apoptosis and other phenotypes by alleviating oxidative stress damage, but whether this protective effect can delay aging is still unknown.

This study has several limitations. First, patients with lumbar degenerative diseases often have severe IDD, making it easy to collect these disc tissues during surgery. However, for healthy patients with no disc degeneration or mild degeneration, doctors cannot obtain disc tissue. This factor results in very few normal intervertebral disc tissues, limiting the sample size of the dataset. Additionally, we found that the sequencing platforms of these datasets are inconsistent, making it impossible to merge the datasets. The limited sample size to some extent reduces the reliability of the results. In the future, we need to continue collecting intervertebral disc tissues from various patients to expand the sample size of the dataset. Second, this study only validated the expression levels of key genes in degenerated NP tissue, but whether these two genes can regulate NPC aging and IDD remains unknown. We speculated that IGFBP3 may have a promoting effect on inflammation, oxidative stress, and cellular senescence within the intervertebral disc. However, given NQO1’s ability to alleviate oxidative stress damage, regulate cell cycle progression, and counteract cell transformation and tumor formation, the increased expression of NQO1 may not be a detrimental factor promoting NPC aging. Therefore, in the upcoming research, we need to validate the impact and regulatory mechanisms of key genes on NPC aging and IDD through *in vivo* and *in vitro* studies by knocking down or overexpressing these genes. Finally, there are many factors that contribute to IDD, and the incidence and severity of disc degeneration vary among different populations. In this study, due to the limited sample size of the dataset, we were unable to control for some potential confounding factors. Additionally, the degree of disc degeneration in the degenerated group was not entirely consistent. These factors may have an impact on the expression of senescence-related genes and immune infiltration. If we cannot effectively reduce the impact of these factors, it may significantly decrease the reliability of the results. In the future, as the sample size of the dataset expands, we need to focus on these confounding factors in the analysis process to mitigate their effects.

In summary, senescence-related genes play an important role in regulating the senescence of NPCs, ECM metabolism, and senescence-related secretory phenotype. The senescence of NPCs is closely related to immune infiltration, which can jointly promote the occurrence of IDD. Through a series of bioinformatics analyses, we have identified for the first time that IGFBP3 and NQO1 may play a central role in the regulation of senescence of NPCs and immune infiltration. The findings of this study provide new insights and important evidence for further elucidating the mechanisms of IDD, and also offer new targets for the treatment of IDD.

## Data Availability

The original contributions presented in the study are included in the article/**Supplementary Material**. Further inquiries can be directed to the corresponding authors.
