# Supplementary material for: Identification of cellular senescence-related genes and immune cell infiltration characteristics in intervertebral disc degeneration
Source: Front Immunol. 2024 Sep 12;15:1439976. doi: 10.3389/fimmu.2024.1439976 (PMC11424418; doi:10.3389/fimmu.2024.1439976)
Supplement: Supplementary file 1 [file Table1.docx]

Supplementary Material

| **Supplementary Table S1. Primers of genes.** | | |
| --- | --- | --- |
| **Gene** |  | **Sequence (5'-3')** |
| IGFBP3 | Forward | CTACGAGTCTCAGAGCACAGATACC |
|  | Reverse | TTTCTCTACGGCAGGGACCATATTC |
| NQO1 | Forward | AGCCGCAGACCTTGTGATATTCC |
|  | Reverse | ATGGCAGCGTAAGTGTAAGCAAAC |
| GAPDH | Forward | TGCCCCCATGTTCGTCA |
|  | Reverse | TTGGCCAGGGGTGCTAAG |
