# Supplementary material for: Identification of cellular senescence-related genes and immune cell infiltration characteristics in intervertebral disc degeneration
Source: Front Immunol. 2024 Sep 12;15:1439976. doi: 10.3389/fimmu.2024.1439976 (PMC11424418; doi:10.3389/fimmu.2024.1439976)
Supplement: Supplementary file 9 [file Table9.docx]

Supplementary Material

| **Supplementary Table S9. Human disc samples from 6 patients** | | | | | |
| --- | --- | --- | --- | --- | --- |
| **Human disc samples** | **Sex** | **Age** | **Diagnosis** | **level** | **Pfirrmann level** |
| 1 | Female | 16 | Isthmic spondylolisthesis | L5/S1 | II |
| 2 | Male | 31 | Lumbar disc herniation | L5/S1 | II |
| 3 | Female | 18 | Isthmic spondylolisthesis | L5/S1 | II |
| 4 | Female | 51 | Lumbar disc herniation | L5/S1 | Ⅳ |
| 5 | Male | 62 | Lumbar disc herniation | L5/S1 | Ⅳ |
| 6 | Female | 55 | Isthmic spondylolisthesis | L5/S1 | Ⅳ |
